# Improvement in Injection Molding Quality Performance with Innovative Cyclone Mixers Used in Polypropylene with Spherical Silicon Dioxide Composites

**DOI:** 10.3390/polym14224932

**Published:** 2022-11-15

**Authors:** Zhan-Xiang Hu, Chang-Chiun Huang, Amit Kumar Gope, Chung-Feng Jeffrey Kuo

**Affiliations:** Department of Materials Science and Engineering, National Taiwan University of Science and Technology, Taipei 10607, Taiwan

**Keywords:** polypropylene, spherical silicon dioxide, principal components analysis, Taguchi method, MPCAC

## Abstract

This research proposes an innovative design of a new cyclone mixer for the quality of polymer materials, and it presents a systematic optimization model of process parameters for plastic injection molding. Thermo gravimetric analysis (TGA) and differential scanning calorimetry (DSC) were used to determine the appropriate thermal properties of processing in order to select appropriate control factors and level values for a Taguchi orthogonal array. The injection molding machine was used to make sample test pieces for tensile strength, hardness and impact strength. Significant factors were found by the signal-to-noise (S/N) ratio with an analysis of variation (ANOVA), and the single-quality optimal parameter combination was obtained. The reproducibility of the experiment was evaluated, and various quality weights were evaluated by principal components analysis (PCA). The multi-quality optimal parameter combination was found, and the comprehensive scores were compared. Finally, the process capability indices were combined with a multi-process capability analysis chart (MPCAC) to compare the process yields of cyclone mixing and screw mixing. The mechanical properties of products were evaluated to verify the performance of cyclone mixing and to provide perfect information for the injection molding quality performance of cyclone mixing and screw mixing. It was concluded that the overall quality of the cyclone mixing products is 42.72, and the total quality of the screw mixing products is 41.85. The total number of defects for the cyclone mixing is 9659 ppm, and that of the screw mixing is 10688 ppm. It can be seen that, for the overall product quality performance, cyclone mixing can be applied in the plastic injection molding process instead of screw mixing.

## 1. Introduction

The development of a polymer blend is often an excellent alternative to reach the desired properties [1,2]. Polymer blends are basically processed by mixing and molding two main steps. Mixing is usually prepared by mechanical mixing in the melted state, such as with the twin screw extruder. The screw-type mixing method involves heating the mold cavity to melt the material, extruding the material through the die and then cooling and cutting it into pellets after mixing, which requires a lot of mixing time and material consumption. This study innovates a rotary mixer instead of using a conventional twin screw extruder in the mixing process to premix various raw materials. This type of cyclone mixer can be directly placed on the injection molding machine, and the raw materials are directly mixed and then sent to the injection molding machine. Mixing and injection are combined to form integrated polymer processing.

Inorganic fillers are widely used in the polymer industry to improve the mechanical properties of polymers. Compared with conventional composites based on micron-sized inorganic fillers, nanocomposites based on nanoscale fillers, such as silica, have attracted great attention because of their improved mechanical properties [3]. To achieve this improvement, nanoscale fillers must be fully dispersed throughout the matrix after using an appropriate modifier to increase their hydrophobicity [3,4]. In this paper, the performance of this integrated polymer processing is verified by the application of polypropylene/silicon dioxide nanocomposites.

### 1.1. Parameter Optimization System

For the PP composite injection molding process, control factors are chosen for some level values and are substituted in the Taguchi method for specific mechanical properties. Zdiri et al. [5] doped low-content nanoparticles to improve the thermal stability and rheological behavior of the original PP, and the mechanical properties of the nanocomposite materials were enhanced. Nakamura et al. [6] indicated that the breaking strength of particle-filled brittle polymer is influenced by the quantity and size of nanoparticles, according to SEM observations on the fracture surface. Wu et al. [7] studied the effect of 3 wt% SiO_2_ surface treatment on the microstructure and particle distribution of PP/SiO_2_ composite and found that a small amount of nano-SiO_2_ can enhance the strength and modulus of the composite effectively. Zheng et al. [8] employed 3 wt% SiO_2_ nanocomposite material to increase the impact strength of nanocomposite material. Gong et al. [9] indicated that 3 wt% PP/SiO_2_ has a higher tensile strength and modulus than other composites. Liang et al. [10] added 2 wt% nano-SiO_2_ in pure PP, and the impact strength was 1.4~1.9 times that of pure PP. Mastali and Dalvand [11] indicated that 1% nano-SiO_2_ particles can enhance the impact resistance and mechanical properties of fibers. Pinto et al. [12] adopted 5 wt% PP/SiO_2_ composite and found that the SiO_2_-doped fiber had good mechanical properties.

The above documents indicate that the addition of SiO_2_ can enhance the mechanical properties of PP, and the optimal content is 1~5% [7,8,9,10,11,12]. Therefore, in this study, the proportions of SiO_2_ are set to 1%, 3% and 5%. 

After the material levels and experimental control factors are selected, an appropriate orthogonal array is selected by using the Taguchi method to plan the experiments in order to greatly reduce the number of experiments [13,14,15]. The contribution degree of the control factors is calculated with the S/N ratio and an ANOVA to find the single-quality optimal factor level combination. With the single-quality optimal factor level combination, the overall quality optimal condition can be explored to ensure good performance of multiple product qualities. According to Liao [16], the Taguchi method cannot currently solve the constant multi-quality response problem, so PCA [13] was combined with the Taguchi method to find the multi-quality response problem. Su and Tong [17] proposed a procedure based on PCA, which can effectively optimize the multi-quality response problem with the Taguchi method. Venkatanarayana et al. [18] combined the Taguchi method with PCA to optimize experimental parameters. Single-quality objective optimization was found by the Taguchi method, and the transformation of a single-quality objective into a multi-quality objective optimization problem was solved by PCA. When combining the Taguchi method with PCA, Sutono et al. [19] uncovered that the complexity trade-off problem in the multi-quality optimization decision process could be solved. 

As the literature in recent years indicates that the Taguchi method, in combination with PCA, can effectively find multi-quality optimal conditions, this study combines the Taguchi method with the PCA method to explore multi-quality optimal conditions. 

### 1.2. Process Capability Indices 

The proposal of the concept of C_p_ of process capability indices (PCIs) [20] by Juran [21] initiated academic research. Kane [22] proposed the one-sided specification process capabilities of C_pl_ and C_pu_ for measuring process variation and proposed the C_pk_ index. The C_pk_ index can reflect the degree of process deviation, but it cannot reflect the situation when the process has variation [23]. Therefore, a new C_pm_ of PCIs was developed according to the concept of the Taguchi loss function proposed by Chan et al. [24]. The C_pm_, also known as the Taguchi index, is a PCI and is closely related to the “Signal to Noise Ratio” concept. The C_pm_ index couples the rate of change of the measured value deviating from the target value with a measurement. As the conditions of process deviation and process variation can be considered simultaneously, the C_pm_ index reflects the degree of process variation better than the C_pk_ index. However, most documents about process capability analysis lay emphasis on a single-quality characteristic [22,24]. There are multiple-quality characteristics for most products in general [20], including the quality characteristics of several one-sided specifications and several double-sided specifications. Each quality characteristic must reach the expected process capability for the customer to accept the product. 

Chen et al. [25] developed a process capability analysis chart (PCAC) for evaluating the capability of a processed product and for providing improvement suggestions for the manufacturing process. Huang and Chen [26] developed a multi-process capability analysis chart (MPCAC) of multiple-quality characteristics for PCI, which is used extensively [27,28]. This study combines the process capability index (C_pm_) with an MPCAC to study the process capabilities of various important quality characteristics, and it develops a new process capability chart called MPCAC/C_pm_, as referred to by Chen et al. [29]. The equipment was designed for analyzing whether the important product quality of cyclone mixing and screw mixing meets the overall process level standard and for ensuring that the process performance meets the product yield requirement [30]. For important product qualities that fall short of the standard, a characteristic diagram influencing the process quality is proposed to improve the process. 

## 2. Materials 

### 2.1. Polypropylene (PP)

The PP for this experiment is Globalene PC366-3, produced by LCY Chemical Corp., Taiwan. Its density is 0.903 (g/cm^3^), the melt flow index is 3 (g/10 min), the tensile strength is 370 (kg/cm^2^), the Rockwell hardness is 98 (R scale), the Izod impact strength is 3 (kg-cm/cm) and the melting point is 160 °C. It is a semi-crystalline polymer that has excellent mechanical properties due to its high crystallinity, including high stiffness, high tensile strength and good thermal deformation resistance. Moreover, the product molding cycle is shortened for its good flow ability and high crystallization temperature. PP is extensively used in different machine shaping techniques, such as injection.

### 2.2. SiO_2_

The spherical SiO_2_ in this experiment is SiO-010-A1 from Fnami Technology Corp. It is characterized by high heat resistance, high moisture resistance, a high dielectric coefficient, high loading, low swelling, low stress, low impurity and a low friction coefficient.

### 2.3. Material Analysis

**Figure 1:**
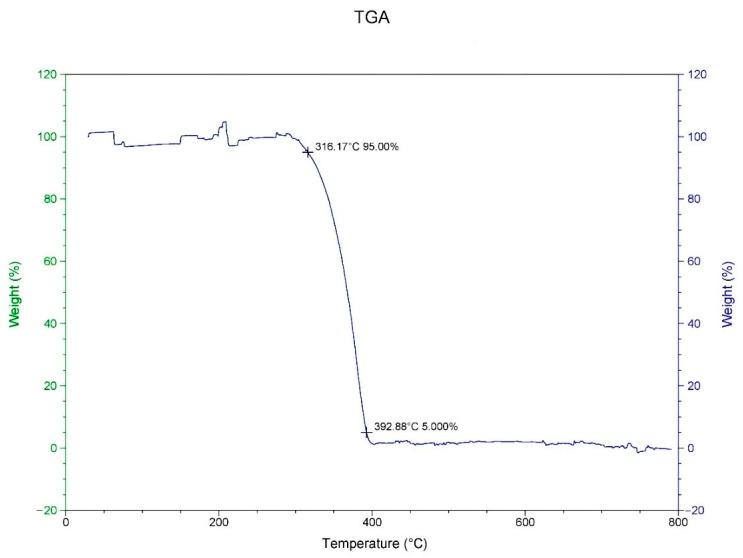
Analysis of thermo gravimetric loss of PP.

In this study, the materials PP and SiO_2_ were analyzed through TGA and DSC to determine the appropriate processing thermal properties and to select appropriate factors in the Taguchi orthogonal table. Figure 1 displays the TGA results from the study of PP. The temperature drop curve was used to determine the pyrolysis temperature. When the volume loss was 5%, the temperature was 316.17 °C, and when it was 95%, the temperature was 392.88 °C.

**Figure 2:**
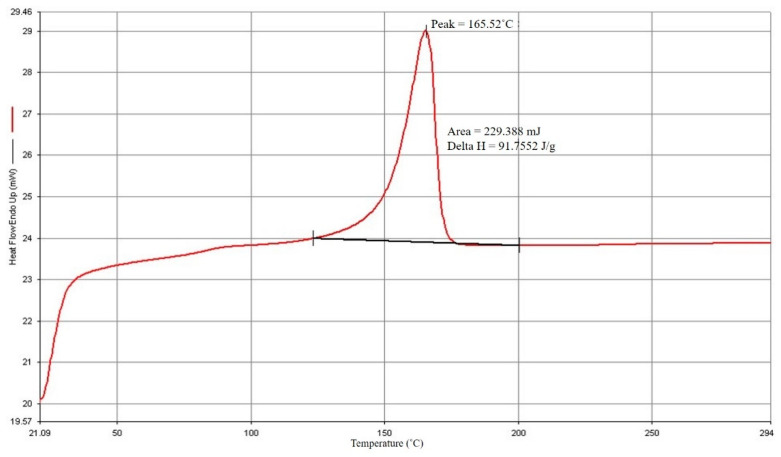
PP melting point.

**Figure 3:**
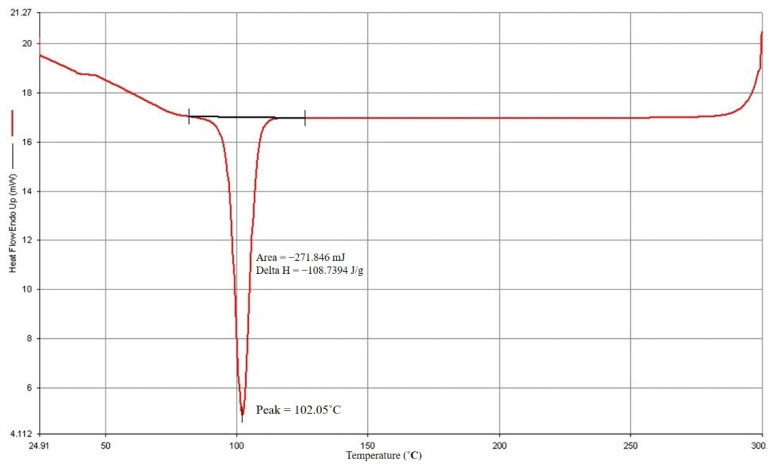
PP crystallization point.

The findings of the DSC study are displayed in Figure 2 and Figure 3. PP has a 165.52 °C melting point and a 102.05 °C crystallization point. The manufacturer and experts advise that the processing temperature should be adjusted to increase the melting temperature by 20° C to 30° C based on the melting point of PP. From this investigation, the starting processing temperature parameter was set to 190° C.

**Figure 4:**
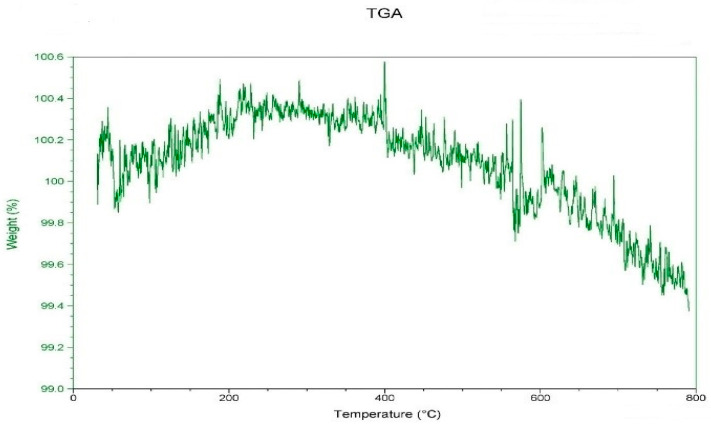
Thermo gravimetric loss of SiO_2_.

**Figure 5:**
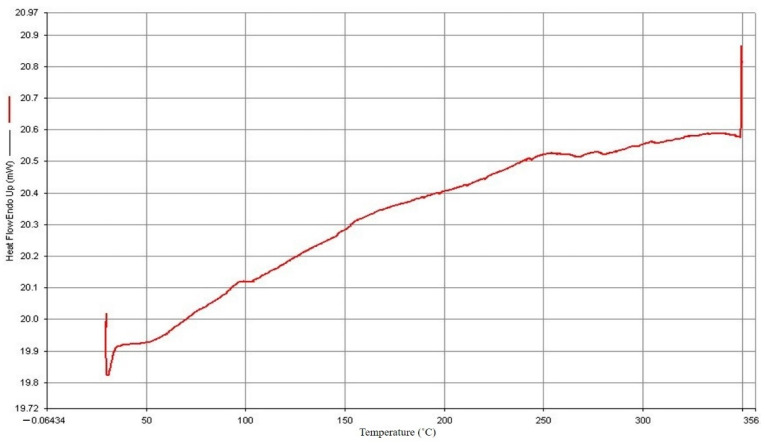
Thermal differential analysis of SiO_2_.

Figure 4 displays the SiO_2_ powder from the TGA research. It was observed that SiO_2_ only lost 0.6 weight percent when heated from 0 °C to 800 °C. When the processing temperature was below 800 °C, it did not initiate thermal cracking. The thermal characteristics of SiO_2_ were attained with the least amount of material weight loss when the highest limit of the DSC heating temperature was, as shown in Figure 5, set to 360 °C. Due to the high pyrolysis temperature, it was difficult to attain the melting temperature. The working temperature was selected to be based on the polymer material PP’s melting point temperature and was raised by about 20 °C; therefore, it could maintain the chemical integrity of PP after mixing with SiO_2_.

## 3. Experimental Machines and Test Equipment

### 3.1. Cyclone Mixer

**Figure 6:**
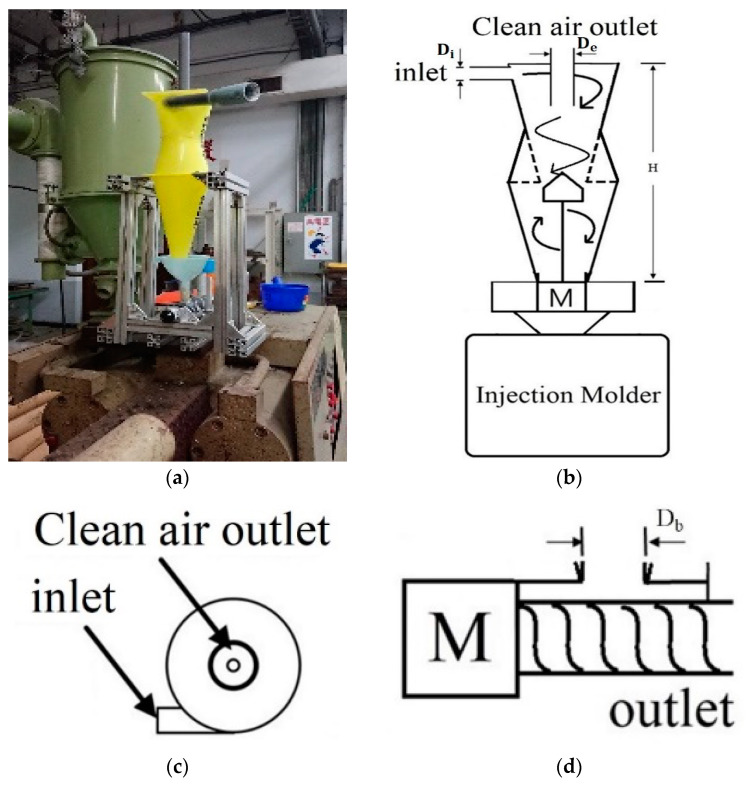
Cyclone mixing: (**a**) Cyclone mixer. (**b**) Cyclone mixer structure diagram. (**c**) Top view of feed inlet. (**d**) Side view of motor.

The operating principle is that the material is rotationally stirred with high-speed airflow to thoroughly mix the material, as shown in Figure 6. The material suction inlet is on the side, the clean air outlet is at the top and the mixed plastic is collected in the lower part and is firmly delivered by the motor and screw to the injection molding machine. The cone tapers downwards; therefore, the original material can move along with the tank wall to the lower area more accurately, and the spiral stroke of the original material in the container is more regular and stable. The cyclone mixer whirls the air in the tank at a high speed, which can drive plastic material and powder material to swirl at a high speed. This principle was employed with the evacuating equipment to provide high efficiency vacuum pumping. After high-speed rotary mixing in the cone container device, the cone-shaped design of the outlet bottom increased the probability of the original material falling into the lower storage tank and reduced the probability of the original material entering the vacuum extractor. By doing so, the lifetime of the vacuumizer and the collection efficiency of the storage tank were improved. To avoid an insufficient rotation period resulting in a poor mixing effect, a cone was added in the middle of the lower portal to the storage vat. This cone inhibited under-mixing the raw material and provided the opportunity of repeatedly stirring the raw material, resulting in enhanced mixing uniformity. The high rotation speed and the discharge screw in the lower part of the storage vat further enhanced the stability of the feed rate for the injection molding machine. The cyclone mixer could be directly placed on the injection molding machine, and mixing and injection were combined to form an integral process. The cyclone mixer is characterized by its compactness, easy installation and ease of use.

### 3.2. Twin Screw Mixer

**Figure 7:**
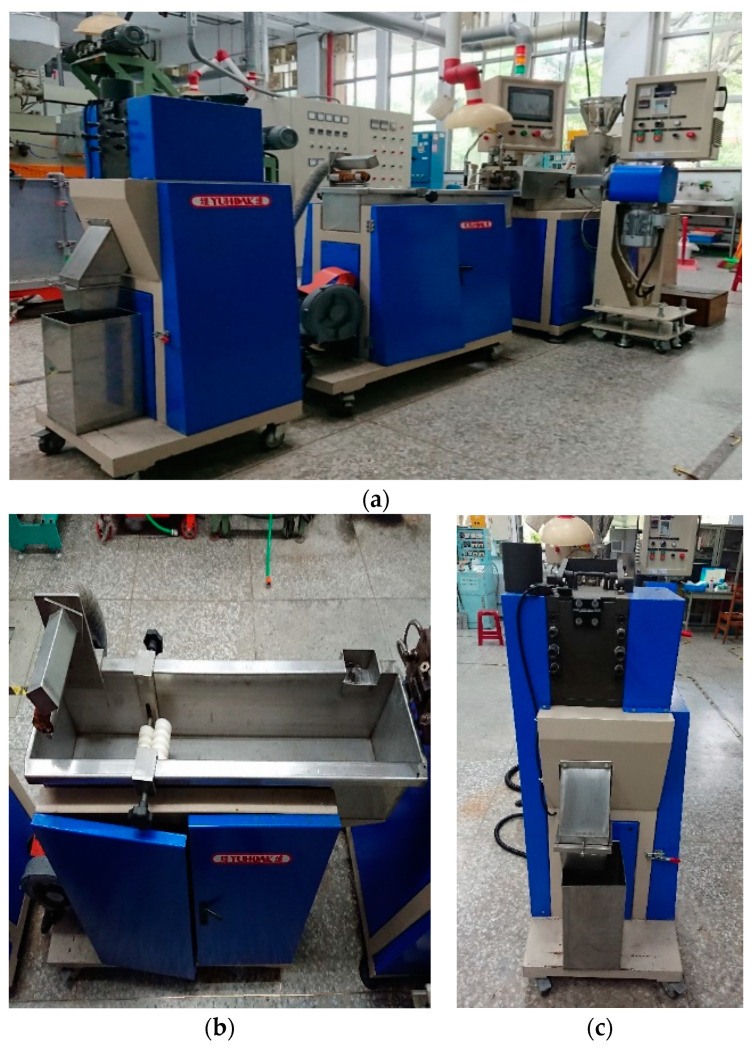
Twin screw mixer: (**a**) Melting and heating zone. (**b**) Water immersion cooling zone. (**c**) Pelletizing and collecting area.

**Figure 8:**
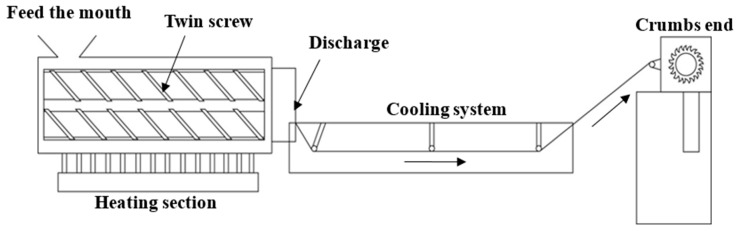
Twin-screw mixer processing procedure.

The twin screw mixer, as shown in Figure 7, was from T-BEG-02042 of Yuh-Dak Machinery Co., Ltd., New Taipei City, Taiwan. Its granulation process is separated into three sections: the melting and heating zone, as shown in Figure 7a; the cooling zone, as shown in Figure 7b; and the pelletizing and collection zone, as shown in Figure 7c. The twin screw mixer processing procedure is shown in Figure 8, and it is described as follows:

- Feeding port: Material is supplied into the feeding hopper through the feeding port.
- Heating Section: The equipment is utilized to heat the thirteen components of the study at the heating portion, where the molten material is combined.
- Discharge port: The molten material is extruded from this, and the equipment utilized in this investigation has a circular section configured as the output.
- Cooling System: The material is cooled down by the water cooling channel after mixing and is then ready for cutting.
- Grain Ends: The substance is sliced into grainy pieces.

The processing parameters were set up according to the material characteristics of PP/SiO_2_. The feed pipe temperature was 190 °C, the feed rate of the feeding machine was 4 Hz, the extrusion rate of the extruder was 8.5 Hz, the chopping speed of the strand cutter was 5 Hz and the SiO_2_ contents were 1%, 3% and 5%. 

**Figure 9:**
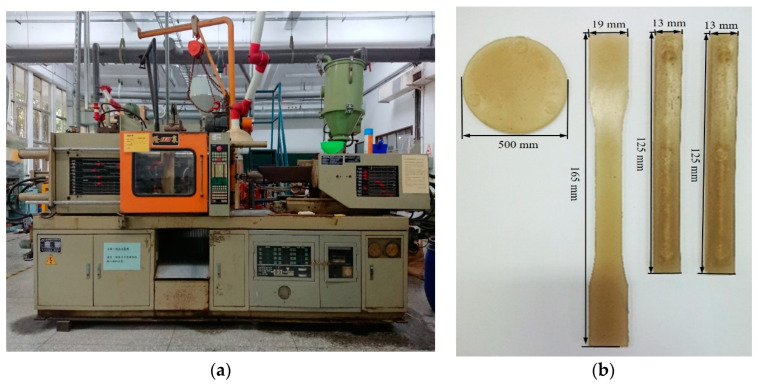
Plastic injection molding machine: (**a**) Plastic injection molding machine. (**b**) Injection molding samples.

The injection molding machine was used mainly for thermoplastic material processing. The injection molding process was divided into heating, filling, packing and cooling stages. By heating the plastic particles in the pipe and rubbing the particles on the pipe wall, the plastic particles could absorb adequate heat energy and were mixed in the molten state. Model LCH-90A of Launch Machinery Works Co., Ltd., New Taipei City, Taiwan, was employed, as shown in Figure 9a. The injection samples are shown in Figure 9b. 

The machining process of the injection molding machine includes heating, filling, pressure retaining and cooling. The plastic material and filler are dried in the oven and are then placed in the feed inlet of the injection molding machine, and the feed rate is controlled. When the particles go in the front end of the feed pipe, the screw drives the material particles to be molten and mixed. The screw thread narrows, the plastic particles and filler are influenced by the temperature inside the pipe and the friction between the particles and the pipe wall, the material particles absorb adequate heat energy and they reach the molten state. The colloid in the molten state is injected through the nozzle into the cooler cavity, and the back pressure and shot pressure are adjusted properly until all the melt goes in the model. This stage is known as the filling stage. As the bonding structure of plastic material is likely to change, high pressure is required inside the model before stabilization to avoid the sample size changing. This stage is known as the packing pressure stage. The final stage is the cooling stage, and the dimensional accuracy and stability of the sample are maintained by adjusting the time. 

According to the optimization of the injection molding process parameters in [15], the process parameters are set, and the feeding speeds are 40 rpm and 60 rpm; the SiO_2_ content is 1%, 3% and 5%; the melting point is 170 °C, 190 °C and 210 °C; the packing pressure is 40 kg/cm^2^, 60 kg/cm^2^ and 80 kg/cm^2^; the dwell time is 0.5 s, 1 s and 1.5 s; the injection pressure is 40 kg/cm^2^, 60 kg/cm^2^ and 80 kg/cm^2^; the injection speed is 30 mm/s, 40 mm/s and 50 mm/s; and the cooling times are 10 s, 12.5 s and 15 s, as shown in Table 1.

### 3.3. Floor Type Dynamic Material Testing Machine 

This study used the MTS-810 floor-type dynamic material testing machine of Sinodynamics Enterprise Co., Ltd., Taipei, Taiwan, for the tensile tests. The sample size conformed to the standard of ASTM D638-14, with the overall width = 19 mm, the width of the narrow section = 13 mm, the length of the narrow section = 57 mm and the overall length = 165 mm, to analyze the polymer quality optimization. 

### 3.4. Shore Hardness Tester 

The model of the hardness tester was D-type. The service condition was that the A-type was used under D20, and the D-type was used above A90. 

### 3.5. Izod Impact Tester 

The izod impact tester was SP-74010 from Yasuda Seiki Seisakusho, Ltd., Hyogo, Japan. The impact test was implemented according to the ISO 180 dimensional standard specifications of 80 × 10 × 4 mm, a pendulum weight of 1.5 kg, a turning radius of 0.42 m and a gap of 45°.

## 4. Research Method and Process Optimization

**Figure 10:**
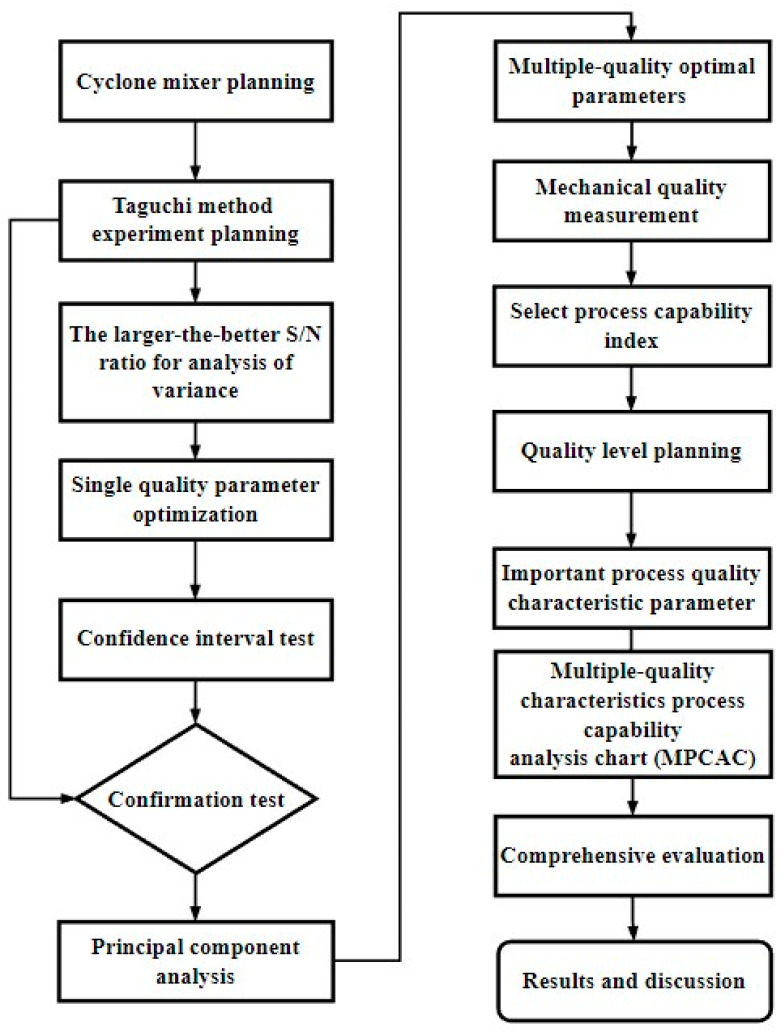
Research flow chart.

The research flow chart is shown in Figure 10. The process capability index Cpm and multiple process capability analysis charts were applied to determine whether cyclone mixing is effective.

### 4.1. Taguchi Quality Engineering 

This experiment required a higher quality and employed the larger-the-better signal-to-noise (S/N) ratio, as expressed by Equation (1).

(1) $$S/N\;\mathrm{ratio}=-10\;\log\left(\frac{\sum_{i=1}^{n}\frac{1}{y_{i}{}^{2}}}{n}\right)$$

where y_i_ is the measured quality value, and n is the number of measured groups.

This experiment adopted the melting temperature, packing pressure, packing time, shot pressure, injection speed, cooling time, feed rate and additive SiO_2_ to form eight factors, including one group of two-level processing parameters and seven groups of three-level processing parameters. The number of factors and level values employed a L_18_ (2^1^ × 3^7^) orthogonal array, and the modified control factor levels are shown in Table 2. 

#### 4.1.1. Analysis of Variance (ANOVA)

The values obtained from the orthogonal array were made into an experimental data sheet, and the variation analysis sheet was made by using the S/N ratio and factor response table.

1. Experimental error S

The square root was taken after taking the sum of the squares of the values in the variation vector, which were divided by their DOF, as expressed in Equation (2).

(2) $$S=\sqrt{\frac{\sum_{i=1}^{r}{\left(y_{i}-\bar{y}\right)}^{2}}{r-1}}$$

where r is the number of measurements, y_i_ is the measured value of machine quality and $\bar{y}$ is the mean value of the measured values.

2. Sum of Squares (SS)

As various groups of data were measured independently, the DOF of the vector was n × r. The mean value of the measured total mechanical quality only contained one $\bar{y}$ value, and the sum of squares was n × r × ${\bar{y}}^{2}$. ${\mathrm{SS}}_{T}$ is expressed as Equation (3).

(3) $${\mathrm{SS}}_{T}=\left(\overset{n}{\underset{i=1}{\sum }}\overset{r}{\underset{j=1}{\sum }}y_{\mathrm{ij}}{}^{2}\right){-n\times r\times \bar{y}}^{2}$$

where n is the number of experimental groups, and r is the number of measurements.

3. Degrees of Freedom (DOF)

The DOF_T_, DOF_A_ and DOF_e_ represent the total variation vector, the effect vector of Factor A and the DOF of the error vector, respectively, as expressed by Equation (4).

(4) $${\mathrm{DOF}}_{T}{=\mathrm{DOF}}_{A}{+\mathrm{DOF}}_{B}{+\mathrm{DOF}}_{C}{+\ldots +\mathrm{DOF}}_{e}$$

4. Average response value estimated variance

The factor variation vector SS_i_ is divided by DOF_i_, as expressed by Equation (5).

(5) $${\mathrm{Var}}_{i\;}=\frac{{\mathrm{SS}}_{i}}{{\mathrm{DOF}}_{i}}$$

5. F Distribution

(6) $$F_{i}=\frac{{\mathrm{Var}}_{i}}{{\mathrm{Var}}_{e}}$$

where Var_e_ is the error of variance Var_e_ estimated by the original sample number, and Var_i_ is the variance of the original sample estimated by the sample mean value. 

#### 4.1.2. Confidence Intervals and Confirmation Experiment

The S/N ratio prediction value is expressed as Equation (7). The confidence limits are the upper bound value and lower bound value of the confidence interval, as expressed in Equation (8), where η is the mechanical quality S/N ratio prediction value, S is the standard deviation, m is the number of samples, TINV is the t-distribution function, α is the confidence level and dof is the DOF of computing the standard deviation. The confidence interval (CI) is α%, as expressed in Equation (9), where η_predict_ is the prediction value, $m_{e}$ is the number of equivalent samples, $m_{r}$ is the number of measured samples and S is the standard deviation. The CI of this experiment is 95%.

(7) $$\eta_{\mathrm{predict}}=\overset{n}{\underset{i=1}{\sum }}\eta_{i}-(n-1)\eta$$

(8) $${\mathrm{CL}}_{\mathrm{predict}\;}{=\eta }_{\mathrm{predict}}\text{±}\frac{S}{\sqrt{m}}\times \mathrm{TINV}(1-\alpha \%,\;\mathrm{dof})$$

(9) $$\mathrm{CI}=\text{±}\sqrt{\frac{S^{2}}{m_{e}}+\frac{S^{2}}{m_{r}}}\times \mathrm{TINV}(1\;-\;\alpha \%,\;\mathrm{dof})$$

### 4.2. PCA Optimization Theory

The quality data were compiled, and Equation (10) was used for standardization processing, where S_i_ is the standardized value of the S/N ratio quality data, x_i_ is the measured quality data, u is the average value of the quality data and σ is the standard deviation.

The eigenvalues of the matrix of the correlation coefficient and the corresponding eigenvector were calculated with Equation (11) to calculate the total score. The principal component scores of various groups of experimental data were added up, as expressed by Equation (12), to obtain the weighted average comprehensive indicator. PC1 is the first principal component, and λ_1_ is the feature value of the first principal component.

(10) $$S_{i}=\frac{x_{i}-u}{\sigma };\;i=1,2,\text{…},n$$

(11) $$\rho_{\mathrm{xy}}=\frac{\sum \left(x_{i}-\bar{x}\right)\left(y_{i}-\bar{y}\right)}{\sqrt{\sum {\left(x_{i}-\bar{x}\right)}^{2}}\sqrt{\sum {\left(y_{i}-\bar{y}\right)}^{2}}}$$

(12) $$\mathrm{Comprehensive}\;\mathrm{score}\;\mathrm{coefficient}=\frac{\lambda_{1}{\times \mathrm{PC}1}_{1}{+\lambda }_{2}{\times \mathrm{PC}2}_{1}{+\lambda }_{3}{\times \mathrm{PC}3}_{1}}{\lambda_{1}{+\lambda }_{2\;}{+\lambda }_{3}}$$

### 4.3. C_pm_ Process Capability Index 

The process capability index C_pm_ is used to combine the variations of off-target values of the measured values, as expressed in Equation (13).

(13) $$C_{\mathrm{pm}}=\frac{\mathrm{USL}-\mathrm{LSL}}{6\sqrt{\sigma^{2}+{\left(u-T\right)}^{2}}}$$

where USL and LSL are the upper and lower limits of specification, u is the process mean, σ is the standard deviation of the process and T is the target value.

### 4.4. Relationship between the C_pm_ Index and the Process Yield 

The process capability C_pmj_, as shown in Equation (14), reflects the precision and precision index simultaneously to evaluate the relatively accurate process capability. The relationship between the process yield $P_{j}$ and C_pmj_ of the process capability index (PCI) is expressed as Equation (15), where Φ is the standard normal distribution function, and P_j_ represents the process yield of the number j of important quality characteristics. As C_pmj_ becomes larger, the process yield becomes higher. There are three important process quality characteristics, which are tensile strength, impact strength and hardness, and the relation between the product yield of the process and the three important process quality characteristics is expressed as Equation (16).

(14) $$C_{\mathrm{pmj}}=\frac{\mathrm{USL}-\mathrm{LSL}}{6\sqrt{\sigma_{j}{}^{2}+{\left(u_{j}{-T}_{j}\right)}^{2}}}=\frac{d_{j}}{3\sqrt{\sigma_{j}{}^{2}+{\left(u_{j}{-T}_{j}\right)}^{2}}}$$

(15) $$P_{j}\geq 2\Phi ({3C}_{\mathrm{pmj}})-1$$

(16) $$P_{j}\geq \overset{3}{\underset{j=1}{\sum }}P_{j}\;\geq \;\overset{3}{\underset{j=1}{\sum }}[2\Phi \left({3C}_{\mathrm{pmj}}\right)-1]$$

### 4.5. MPCAC

This study develops the process capability analysis chart for evaluating multiple-quality characteristics of plastic injection molding products (MPCAC/C_pm_) according to the C_pm_ evaluation index proposed by Chen et al. [29]. 

The process accuracy index (δ) is defined as Equation (17), and the process precision index (γ) is defined as Equation (18). If the accuracy index ($\delta_{j}$) is used as X-axis and the process precision index ($\gamma_{j}$) is used as Y-axis, the equation C_pmj_ can be changed to Equation (19), meaning that, as the r value becomes smaller, PCI C_pmj_ becomes larger. This also indicates that the process capability accuracy and process capability precision are increased accordingly and thus have better quality.

(17) $$\delta_{j}=\left(\frac{u_{j}{-T}_{j}}{d_{j}}\right),\;j=1,2,3$$

(18) $$\gamma_{j}=\left(\frac{\sigma_{j}}{d_{j}}\right),\;j=1,2,3$$

(19) $$C_{\mathrm{pmj}}=\frac{d_{j}}{3\sqrt{\sigma_{j}{}^{2}+{\left(u_{j}{-T}_{j}\right)}^{2}}}=\frac{1}{3\sqrt{\delta_{j}{}^{2}{+\gamma }_{j}{}^{2}}}=\frac{1}{3\sqrt{r^{2}}}=\frac{1}{3(r)}$$

**Figure 11:**
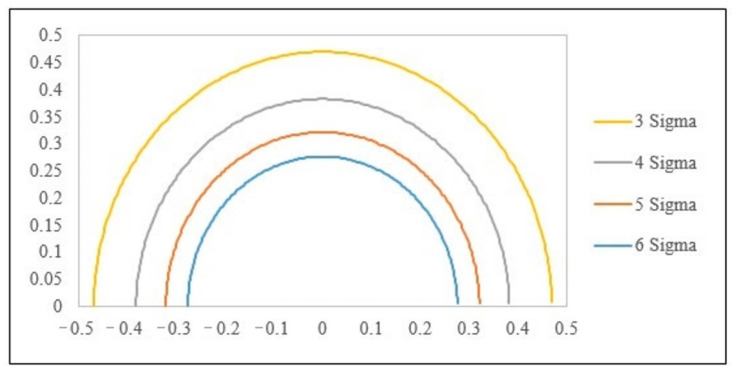
Multiple-quality characteristics process capability analysis chart (MPCAC/C_pm_).

The six standard deviation quality levels proposed by Motorola refer to the process standard σ = d/6 and allow for a process deviation of 1.5σ, i.e., γ_j_ = 1/6 and |δ_j_| ≤ 0.25. According to this condition, taking 6σ as an example, the lower bound value of the corresponding C_pmj_ is C_pmj_ ≥ 1.207. The lower bound value of the process index value C_pmj_ of various important quality characteristics in the cases of 5 Sigma, 4 Sigma and 3 Sigma can be determined according to this method. The multiple-quality characteristics process capability analysis chart (MPCAC/C_pm_) is formed, as shown in Figure 11. 

## 5. Result and Discussions

### 5.1. Single-Quality Optimal Experimental Results 

The larger-the-better S/N ratio value was calculated by using Equation (1). In this section, significant parameters which influence the product quality are found, as shown in Table 3. Then, the single-quality optimal parameter group is obtained from the S/N ratio response table. 

The quality S/N ratio values in Table 3 were substituted into Equations (2)–(6). The quality characteristics were used in the variation analysis, and the following significant factors in screw mixing were obtained: the tensile strength is B, C, F and H; the impact strength is B, C, E and F; and the hardness is A, B, C and E. The significant factors in cyclone mixing are as follows: the tensile strength is A, B, C and F; the impact strength is C, E, F and G; and the hardness is A, B, C and H. 

According to the ANOVA of screw mixing tension, the significant factors influencing the mechanical properties include (B) SiO_2_ content, (C) melting temperature, (F) shot pressure and (H) cooling time, with a standard deviation of S = 0.16 and with dof_e_ = 9. The tension S/N ratio prediction value η = 31.523 and the number of equivalent samples m_e_ = 2 were calculated with Equations (7)–(9) with a 95% CI of [−0.28 0.28]. The screw type tension prediction value S/N ratio is 31.523, and the experimental value S/N ratio is 31.526. As the difference is 0.0036, this error is in the 95% confidence interval. The rest can be deduced accordingly, and the results are shown in Table 4. The experimental results show good reproducibility. 

### 5.2. Multi-Quality Parameter Optimization 

This section describes the combination of the Taguchi method and PCA in order to obtain the comprehensive appraisal value of the processing parameters. 

#### 5.2.1. Optimization Parameters of PCA 

Step 1: The S/N ratio data of the three mechanical properties, tension, hardness and the impact of screw mixing and cyclone mixing, are integrated. 

Step 2: The S/N ratio data of each quality are standardized with Equation (10) to obtain the standardized data. 

Step 3: The standardized data are calculated with Equation (11) to find the matrix of the correlation coefficient of various qualities, as shown in Table 5. 

Step 4: The eigenvalues and eigenvectors of the parameters are calculated according to the correlation coefficient in Table 6. The standardized data of the S/N ratio and the eigenvectors are substituted into Equation (12) to obtain the principal component score (PCS) and the total score (TS), as shown in Table 6.

#### 5.2.2. Best Multi-Quality Parameter Combination 

The control factors with a higher score are conditions with significant influence, and the combination of the optimal conditions is the multi-quality optimal parameter group. The results are shown in Table 7. Screw mixing is A1, B3, C1, D3, E1, F2, G3 and H3, which are a feed rate of 40 rpm, a SiO_2_ content of 5%, a melting temperature of 170 °C, a packing pressure of 80 kg/cm^2^, a packing time of 0.5 s, a shot pressure of 60 kg/cm^2^, an injection speed of 50 mm/s and a cooling time of 15 s. Cyclone mixing is A2, B2, C1, D1, E3, F1, G1 and H1, which are a feed rate of 60 rpm, a SiO_2_ content of 3%, a melting temperature of 170 °C, a packing pressure of 40 kg/cm^2^, (E) a packing time of 1.5 s, (F) a shot pressure of 40 kg/cm^2^, (G) an injection speed of 30 mm/s and a cooling time of 10 s.

#### 5.2.3. Experimental Results of Optimization Parameters 

Afterwards, the optimization parameters were adopted in the experiment, as shown in Table 8. 

To measure the influence of the multi-quality parameters on the process, PCA can effectively find the weight ratio of each variation, calculate the quality variation contributions and comprehensive score coefficients and find an effective correlation of various process qualities when calculating multivariable data, which is a method used to measure the weight of the multi-quality parameters. The quality variation contributions and the rotated component matrix of cyclone mixing and screw mixing under multi-quality parameters were calculated by PCA. The relationship between the eigenvalues and the variance was calculated with Equation (12) to find the linear combination coefficients of various principal components, as shown in Table 9.

After summing the linear coefficients and eigenvalues of the principal components, the comprehensive weight ratio was obtained, as shown in Table 10. The S/N ratio of the multi-quality optimal process was integrated by using the quality weight ratio, and the score of a comprehensive appraisal of screw mixing and cyclone mixing was obtained, as shown in Table 11. The overall score of cyclone mixing was 42.72, and the overall score of screw mixing was 41.85, meaning that cyclone mixing has a better overall mechanical property quality performance than that of screw mixing. 

### 5.3. Process Capability Performance Analysis 

The subsequent product quality stability of cyclone mixing and screw mixing was measured by using the C_pm_ of PCI in this section in order to discuss whether cyclone mixing can maintain a high product yield with a relatively high product quality performance. The target value was regarded as the midpoint of the specification limit [31], and tension, impact and hardness control charts were made using the quality data in Table 11 in order to obtain the upper bound value and lower bound value of each process quality. For the purpose of comparing the process capabilities of cyclone mixing and screw mixing, the multi-quality optimal parameters were employed for the injection molding experiment, and the tensile, impact and hardness tests were performed for the experimental samples. The average value, standard deviation and specification tolerance of quality data were calculated, as shown in Table 12.

#### 5.3.1. Multi-Quality Process Capability Analysis Chart

**Figure 12:**
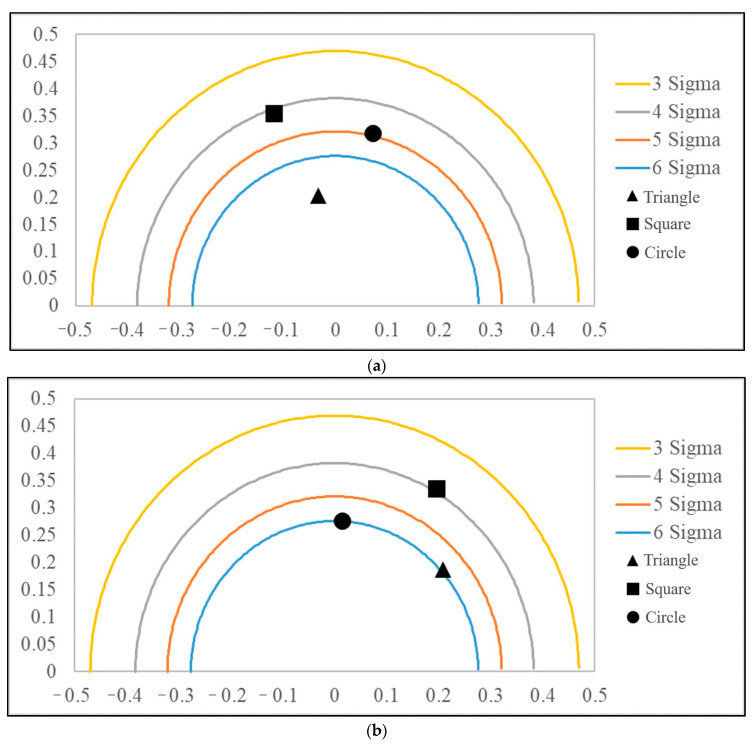
MPCAC/C_pm_ of two types: (**a**) Cyclone type. (**b**) Screw type.

The quality data in Table 13 were substituted into Equations. (17)–(18) to calculate the accuracy and precision values corresponding to the quality data, as shown in Table 14. The accuracy and precision values were adopted as coordinates to draw three important quality level positions in the six-sigma-level contour diagram, as shown in Figure 12.

#### 5.3.2. Multi-Quality Process Capability Analysis 

Each coordinate point (δ, γ) of the cyclone type and the screw type of MPCAC/C_pm_ represents a quality process. This research employs six sigma to analyze the product yield of cyclone mixing and screw mixing, and the instability of the two processes was analyzed by using the yields. The relationships of accuracy and precision to the C_pm_ value are found through Equation (19), and the defect rate corresponding to each important quality is calculated by Equation (15), as shown in Table 14. Afterwards, the numbers of defects and the total number of defects of the two processes are integrated by Equation (16), where that of cyclone mixing is 9659 ppm (parts per million), and that of screw mixing is 10,688 ppm. Comparing the overall quality defect rate of the two processing processes, the defect rate of cyclone mixing is lower than that of screw mixing.

The cyclone mixer can be directly placed on the injection molding machine, and mixing and injection are combined to form an integral process. With the conical shape of cyclone mixing, the air spins fast along the end wall; therefore, the material is collected downwards and is mixed. For screw mixing, the material is molten from cavity heating, extruded through the die orifice, cooled and cut into mixed particles. The twin-screw mixer needs to preheat for 2 h in advance, and then it takes almost an hour for the whole procedure. After that, the pellets are left for a minimum of 8 h to allow them to solidify before being inserted into an injection molding machine. If we consider the total overall mixing and processing time for the twin-screw to be about 11 h, it is a drawn-out and time-consuming procedure for the twin-screw mixer.

For cleaning after mixing, some components of the cyclone mixer, such as the feed pipe, conical cavity and feed screw, are easy to assemble and disassemble and can be detached and cleaned. This is better than conventional screw mixing, which consumes a lot of raw materials for the cleaning step. The cleaning of the cyclone mixer is more complete, and the consumption of raw materials is low.

## 6. Conclusions

A comparison of the injection molding quality performance and process capabilities between cyclone mixers with innovative designs and conventional screw mixing is presented in this study via the application of polypropylene /silicon dioxide composites.

The Taguchi method and the principal component analysis method are combined to find the best multi-quality parameter group. According to the parameters, it can be concluded that the best parameter group of multi-quality screw mixing is as follows: (A) a feeding speed of 40 rpm, (B) a SiO_2_ content of 5%, (C) a melting temperature of 170 °C, (D) a holding pressure 80 kg/cm^2^, (E) a holding time of 0.5 s, (F) an injection pressure of 60 kg/cm^2^, (G) an injection speed of 50 mm/s and (H) a cooling time of 15 s. The best parameter group for multi-quality cyclone mixing is as follows: (A) a feeding speed of 60 rpm, (B) a SiO_2_ content of 3%, (C) a melting temperature of 170 °C, (D) a holding pressure of 40 kg/cm^2^, (E) a holding time of 1.5 s, (F) an injection pressure of 40 kg/cm^2^, (G) an injection speed of 30 mm/s and (H) a cooling time of 10 s. The quality measurement values of the best multi-quality parameter products are all close to the quality measurement values of each best single-quality parameter product. Then, through principal component analysis, three quality weights of impact and hardness are evaluated, and it is concluded that the overall score of cyclone mixing is 42.72 and that the overall score of screw mixing is 41.85. The cyclone mixing has a better overall mechanical property quality performance than that of screw mixing. The total number of defects of cyclone mixing is 9659 ppm (parts per million), and that of screw mixing is 10688 ppm, which shows that the overall product quality performance of cyclone mixing is better than that of screw mixing. The injection molding quality performances and process capabilities between cyclone mixers with innovative designs and conventional screw mixing are presented in this study via the application of polypropylene /silicon dioxide composites.

## Figures and Tables

**Table 1:** Processing parameter conditions.

Factor	Description	Design Value			
1	Feed rate		40	60	rpm
2	SiO_2_ content	1	3	5	%
3	Melting temperature	170	190	210	°C
4	Packing pressure	40	60	80	kg/cm^2^
5	Packing time	0.5	1	1.5	s
6	Shot pressure	40	60	80	kg/cm^2^
7	Injection speed	30	40	50	mm/s
8	Cooling time	10	12.5	15	s

**Table 2:** Experimental parameters of orthogonal array.

Factor	Feed Rate (rpm)	SiO_2_ (%)	Melting Temperature (°C)	Packing Pressure (kg/cm^2^)	Packing Time (s)	Shot Pressure (kg/cm^2^)	Injection Speed (mm/s)	Cooling Time (s)
Level 1	40	1	170	40	0.5	40	30	10
Level 2	60	3	190	60	1	60	40	12.5
Level 3		5	210	80	1.5	80	50	15

**Table 3:** Quality characteristics S/N ratio larger-the-better analysis.

No.	Screw Mixing			Cyclone Mixing		
	Tensile Strength	Hardness	Impact	Tensile Strength	Hardness	Impact
1	31.48	37.31	19.98	31.09	37.40	22.39
2	31.39	37.25	8.50	30.94	37.17	14.51
3	31.36	37.25	15.54	30.70	37.18	15.44
4	30.83	37.30	18.61	30.63	37.27	19.25
5	31.29	37.22	17.29	30.47	37.29	18.45
6	30.94	37.26	11.64	30.72	37.11	11.87
7	31.18	37.32	19.66	30.61	37.22	15.22
8	31.18	37.26	10.15	30.63	37.22	14.46
9	30.72	37.25	16.47	30.51	37.24	16.64
10	31.61	37.14	20.52	30.81	37.15	17.27
11	31.36	37.08	8.19	31.07	37.06	12.86
12	30.94	37.04	6.49	30.97	36.97	12.10
13	31.13	37.28	20.01	31.16	37.27	21.05
14	31.10	37.25	18.34	30.88	37.22	19.93
15	31.11	37.21	11.36	30.76	37.20	12.15
16	31.38	37.39	18.34	30.89	37.31	16.42
17	31.34	37.28	9.33	31.11	37.19	17.58
18	31.01	37.34	15.78	30.31	37.31	14.95

**Table 4:** Experimental values of single-quality optimal parameters e.

Screw Type								
Tensile strength					Experimental S/N ratio	Predicted S/N ratio	Difference	95% CI
37.60	38.08	37.56	37.44	37.62	31.53	31.523	0.0036	±0.28
37.47	37.76	37.72	37.87	37.88				
Hardness								
74.00	74.30	74.40	74.30	74.60	37.40	37.385	0.0177	±0.12
73.40	73.90	74.20	74.00	74.50				
Impact strength								
11.84	11.73	10.96	11.40	12.50	21.47	23.008	1.54	±4.44
12.06	12.06	12.39	11.95	11.73				
**Cyclone Type**								
Tensile strength					Experimental S/N ratio	Predicted S/N ratio	Difference	95% CI
36.66	37.28	37.34	37.33	36.77	31.44	31.25	0.187	±0.26
37.06	37.50	37.95	38.02	37.27				
Hardness								
75.20	74.90	74.80	75.60	74.70	37.48	37.38	0.098	±0.14
74.60	74.90	74.50	74.70	74.50				
Impact strength								
14.02	13.70	13.81	13.59	13.59	22.64	22.33	0.311	±3.41
13.81	13.37	13.27	13.27	13.16				

**Table 5:** Matrix of the correlation coefficient.

Screw Mixing				Cyclone Mixing			
	Tensile Strength	Hardness	Impact Strength		Tensile Strength	Hardness	Impact Strength
Tensile strength	1	−0.049	0.102	Tensile strength	1	−0.229	0.218
Hardness	−0.049	1	0.524	Hardness	−0.229	1	0.707
Impact strength	0.102	0.524	1	Impact strength	0.218	0.707	1

**Table 6:** Principal component score.

Screw Mixing					Cyclone Mixing				
No.	PCS1	PCS2	PCS3	TS	No.	PCS1	PCS2	PCS3	TS
1	−0.036	−1.167	1.359	0.290	1	−0.406	1.180	2.741	1.997
2	1.109	−0.740	−0.854	−0.518	2	−0.306	0.536	−0.698	−0.218
3	0.078	−0.714	0.214	−0.122	3	0.132	−0.349	−0.366	−0.334
4	−0.388	1.541	0.932	0.938	4	0.460	−0.543	1.127	0.470
5	−0.518	−0.544	0.163	−0.180	5	0.381	−1.263	1.103	0.184
6	0.384	1.123	−0.417	0.227	6	−0.167	−0.420	−1.684	−1.135
7	−0.158	0.090	1.295	0.665	7	0.008	−0.794	−0.188	−0.404
8	0.779	0.152	−0.585	−0.128	8	−0.214	−0.820	−0.317	−0.499
9	−0.599	1.894	0.134	0.620	9	0.293	−1.152	0.321	−0.230
10	−1.313	−2.089	0.150	−0.832	10	0.603	0.296	−0.208	0.022
11	−0.212	−0.949	−2.272	−1.510	11	−0.097	1.146	−1.866	−0.652
12	−0.618	0.705	−2.975	−1.369	12	0.445	0.930	−2.643	−1.152
13	−0.515	0.219	1.061	0.536	13	0.059	1.677	1.516	1.501
14	−0.539	0.292	0.554	0.299	14	0.597	0.641	0.878	0.775
15	0.120	0.283	−0.862	−0.324	15	−0.744	−0.452	−1.005	−0.786
16	0.765	−0.568	1.733	0.805	16	−0.773	0.171	0.736	0.448
17	1.180	−0.445	−0.505	−0.229	17	−0.039	1.412	0.126	0.597
18	0.481	0.918	0.876	0.830	18	−0.233	−2.197	0.426	−0.585

**Table 7:** The total principal component score.

Screw Type								
	A	B	C	D	E	F	G	H
	Feed rate	SiO_2_	Melting temperature	Packing pressure	Packing time	Shot pressure	Injection speed	Cooling time
Level 1	0.1993	−0.6768	0.4004	−0.0359	0.1336	−0.1121	0.0777	−0.0574
Level 2	−0.1993	0.2495	−0.3776	−0.0057	−0.0992	0.2231	−0.1685	0.0268
Level 3		0.4273	−0.0228	0.0416	−0.0344	−0.1110	0.0908	0.0306
**Cyclone Type**								
	**A**	**B**	**C**	**D**	**E**	**F**	**G**	**H**
	Feed rate	SiO_2_	Melting temperature	Packing pressure	Packing time	Shot pressure	Injection speed	Cooling time
Level 1	−0.0186	−0.0562	0.6723	0.2328	−0.0006	0.2182	0.3370	0.0554
Level 2	0.0186	0.1684	0.0312	−0.1122	−0.2894	0.0390	−0.3205	−0.0475
Level 3		−0.1122	−0.7035	−0.1205	0.2900	−0.2572	−0.0165	−0.0079

**Table 8:** Multi-quality process parameter optimization data.

Screw Mixing					Cyclone Mixing				
Tensile strength (Mpa)									
36.38	36.26	36.30	36.31	36.25	36.35	36.21	36.52	36.29	36.37
36.36	36.18	36.59	36.36	36.63	36.45	36.18	36.19	36.22	36.54
36.33	36.58	36.41	36.31	36.40	36.45	36.48	36.43	36.46	36.34
36.41	36.41	36.36	36.49	36.20	36.29	36.30	36.31	36.26	36.52
Impact strength (kJ/m^2^)									
10.96	10.85	11.29	10.41	10.19	11.73	12.06	12.28	11.51	11.95
10.63	10.63	10.96	10.19	10.85	12.06	12.17	11.73	11.95	12.17
11.17	10.81	10.71	10.78	10.63	12.16	11.75	11.63	12.07	12.04
10.88	10.83	11.27	10.45	10.68	11.78	11.74	12.15	11.55	12.10
Hardness (Shore D)									
75.10	75.10	75.80	75.00	73.40	74.20	74.10	74.50	73.40	74.20
74.30	74.80	74.60	74.20	73.50	74.20	73.60	73.70	74.70	73.50
74.01	75.37	74.68	75.16	73.93	74.56	73.82	73.52	74.26	74.27
75.43	74.69	73.97	75.24	74.20	74.51	73.45	74.50	73.97	73.69

**Table 9:** Linear combination coefficients of principal components.

Screw Mixing				Cyclone Mixing			
	1	2	3		1	2	3
Tensile strength	0.992	−0.009	0.128	Tensile strength	0.978	−0.017	0.207
Impact strength	−0.009	0.997	0.078	Impact strength	−0.016	0.985	0.172
Hardness	0.129	0.080	0.988	Hardness	0.220	0.187	0.957
Eigenvalue	1.000	1.000	0.999	Eigenvalue	1.006	1.005	0.989
Coefficients in the linear combination							
Tensile strength	0.9916	−0.0087	0.1282	Tensile strength	0.9754	−0.0170	0.2086
Impact strength	−0.0085	0.9968	0.0776	Impact strength	−0.0162	0.9824	0.1729
Hardness	0.1294	0.0796	0.9887	Hardness	0.2199	0.1863	0.9626

**Table 10:** Calculated weight index.

Screw Mixing				Cyclone Mixing			
Comprehensive Score Coefficient		Index Weight		Comprehensive Score Coefficient		Index Weight	
Tensile strength	0.37	Tension	0.329	Tensile strength	0.390	Tension	0.319
Impact strength	0.36	Impact	0.316	Impact strength	0.381	Impact	0.311
Hardness	0.40	Hardness	0.355	Hardness	0.453	Hardness	0.370

**Table 11:** Integrated quality data scores.

Screw Mixing					Cyclone Mixing			
No.	Tensile Strength	Impact Strength	Hardness	Total Score	Tensile Strength	Impact Strength	Hardness	Total Score
1	36.38	10.96	75.10	42.08	36.35	11.73	74.20	42.71
2	36.26	10.85	75.10	42.01	36.21	12.06	74.10	42.73
3	36.30	11.29	75.80	42.41	36.52	12.28	74.50	43.05
4	36.31	10.41	75.00	41.85	36.29	11.51	73.40	42.33
5	36.25	10.19	73.40	41.19	36.37	11.95	74.20	42.79
6	36.36	10.63	74.30	41.69	36.45	12.06	74.20	42.85
7	36.18	10.63	74.80	41.81	36.18	12.17	73.60	42.57
8	36.59	10.96	74.60	41.98	36.19	11.73	73.70	42.48
9	36.36	10.19	74.20	41.51	36.22	11.95	74.70	42.93
10	36.63	10.85	73.50	41.57	36.54	12.17	73.50	42.65
11	36.33	11.17	74.01	41.75	36.45	12.16	74.56	43.01
12	36.58	10.81	75.37	42.20	36.48	11.75	73.82	42.62
13	36.41	10.71	74.68	41.87	36.43	11.63	73.52	42.46
14	36.31	10.78	75.16	42.03	36.46	12.07	74.26	42.88
15	36.40	10.63	73.93	41.57	36.34	12.04	74.27	42.83
16	36.41	10.88	75.43	42.19	36.29	11.78	74.51	42.82
17	36.41	10.83	74.69	41.91	36.30	11.74	73.45	42.42
18	36.36	11.27	73.97	41.78	36.31	12.15	74.50	42.94
19	36.49	10.45	75.24	42.01	36.26	11.55	73.97	42.54
20	36.20	10.68	74.20	41.62	36.52	12.10	73.69	42.69
Ave.	36.38	10.76	74.62	41.85	36.36	11.93	74.03	42.72

**Table 12:** Quality data.

Cyclone Mixing				
	Target value	Average value	Standard deviation	Specification tolerance
Tensile strength	36.36	36.29	0.406	2.001
Impact strength	11.93	11.73	0.592	1.671
Hardness	74.70	74.17	0.735	1.905
**Screw Mixing**				
	Target value	Average value	Standard deviation	Specification tolerance
Tensile strength	36.38	36.76	0.348	1.862
Impact strength	10.76	11.09	0.561	1.672
Hardness	74.62	74.65	0.527	1.899

**Table 13:** Process defect rate.

Screw Mixing			Cyclone Mixing		
	C_pm_ Value	Defect Rate (ppm)		C_PM_ Value	Defect Rate (ppm)
Tensile strength	1.1901	356	Tensile strength	1.6216	1
Impact strength	0.8584	10,015	Impact strength	0.8921	7444
Hardness	1.2003	317	Hardness	1.2120	2214

**Table 14:** Process parameter datasheet of important quality characteristics.

	Cyclone Mixing			Screw Mixing		
Quality Characteristic	Specification	Accuracy	Precision	Specification	Accuracy	Precision
Tensile strength	36.36	−0.0333	0.2028	36.38	0.2087	0.1868
Impact strength	11.93	−0.1185	0.3544	10.76	0.1959	0.3353
Hardness	74.03	0.0696	0.3859	74.62	0.0137	0.2774

## Data Availability

Not applicable.
